# Unrecognised Outbreak: Human parainfluenza virus infections in a pediatric oncology unit.  A new diagnostic PCR and virus monitoring system may allow early detection of future outbreaks

**DOI:** 10.12688/wellcomeopenres.14732.1

**Published:** 2018-09-19

**Authors:** Anna Smielewska, Callum Pearson, Ashley Popay, Iain Roddick, Mark Reacher, Edward Emmott, Jenny He, Rachel Thaxter, Carol Chenery, Ian Goodfellow, Amos Burke, Hamid Jalal

**Affiliations:** 1 Division of Virology, Department of Pathology, University of Cambridge Addenbrooke's Hospital Cambridge, Cambridge, Cambridgeshire, CB2 0QQ, UK; 2 Public Health Laboratory, Cambridge University Hospitals NHS Foundation Trust, Public Health England, Cambridge, Cambridgeshire, CB2 0QQ, UK; 3 Field Epidemiology Service East of England, Public Health England, Cambridge, Cambridgeshire, CB20SR, UK; 4 Department of Bioengineering, Northeastern University, Boston, MA, 02115-5000, USA; 5 Infection Control, Cambridge University Hospitals, NHS Foundation Trust, Cambridge, Cambridgeshire, CB2 0QQ, UK; 6 Department of Paediatric Oncology, Cambridge University Hospitals NHS Foundation Trust, Cambridge, Cambridgeshire, CB2 0QQ, UK

**Keywords:** human parainfluenza 3, outbreak, paediatric, unrecognised, oncology, infection control, diagnostic PCR

## Abstract

**Background: **Human parainfluenza viruses (HPIVs) are significant causes of both upper and lower respiratory tract infections with type 3 (HPIV3) causing the most severe disease in the immunocompromised cohorts.  The objective of this study was to analyse the epidemiological nature of a cluster of cases of HPIV3 in a pediatric oncology unit of a major teaching hospital.

**Methods: **In order to determine whether the activity observed represented a deviation from the norm, seasonal trends of HPIV3 in the surrounding geographical area as well as on the ward in question were analysed.  The genetic link between cases was established by the phylogenetic analysis of the non-coding hypervariable region between the M (Matrix) and F (fusion) genes of HPIV3. The 15 cases involved and 15 unrelated cases were sequenced.  Transmission routes were subsequently inferred and visualized using Konstanz Information Miner (KNIME) 3.3.2.

**Results: **Of the 15 cases identified, 14 were attributed to a point source outbreak. Two out of 14 outbreak cases were found to differ by a single mutation A182C. The outbreak strain was also seen in 1 out of 15 unrelated cases, indicating that it was introduced from the community. Transmission modeling was not able to link all the cases and establish a conclusive chain of transmission. No staff were tested during the outbreak period. No deaths occurred as a result of the outbreak.

**Conclusion: **A point source outbreak of HPIV3 was recognized *post factum* on an oncology pediatric unit in a major teaching hospital. This raised concern about the possibility of a future more serious outbreak. Weaknesses in existing systems were identified and a new dedicated respiratory virus monitoring system introduced.  Pediatric oncology units require sophisticated systems for early identification of potentially life-threatening viral outbreaks.

## Introduction

Human parainfluenza viruses (HPIV) are members of the family *Paramyxoviridae* and are subdivided into four types, which fall into two genera *rubulavirus* (types 2 and 4) and *respirovirus* (types 1 and 3) ^1, 2^. All four types of HPIV are significant causes of both upper and lower respiratory tract infections with human parainfluenza virus type 3 (HPIV3) accounting for the majority of these, and has the highest mortality ^3^. Immunity to HPIV is incomplete and repeated infections occur throughout life ^2^. In England and Wales HPIV3 has been shown to have seasonal late spring and summer peaks with little variation from this pattern reported globally ^4^.

Transmission of HPIV3 is through respiratory droplets and fomites, where the virus can remain viable for up to 10 hours given the right conditions of temperature and humidity ^5^. Extensive studies conducted with respiratory syncytial virus (RSV), a closely related paramyxovirus, have also identified gloves and hospital gowns as potential sources of nosocomial transmission ^6^. Transmission within the immunocompromised cohort is exacerbated by prolonged asymptomatic shedding, although in the pediatric cohort the evidence for this is conflicting ^7, 8^. Pediatric outbreaks are further complicated by complex shared transmission routes involving communal areas, toys and patterns of behavior that involve self-inoculation via eyes, nose and mouth ^6^.

A number of small studies have evaluated the impact of respiratory viruses including HPIV3 in the pediatric oncology cohort ^8– 17^. In each case, outbreaks were identified retrospectively and prospective identification of a point sources has not been possible ^8, 9^. There is limited consensus on prevalence, severity of infection and clinical outcome. HPIV3 has been reported as a causative pathogen in 1–18% of respiratory viral infections in pediatric oncology patients, presenting both as a common, as well as a relatively rare, pathogen ^12, 13, 15– 17^ with conflicting clinical outcome ^9– 14^ and immunosuppression status as a potential confounding factor ^13– 15^. Additionally, the prevalence of infections with more than one respiratory virus in this cohort has been quoted to be between 2 and 26% with both no clinical impact ^14, 17^ and increased severity of infection reported ^13, 18^. The above suggests that dual infections may additionally play an important role in the clinical outcome and the possibility of dual transmission in pediatric outbreaks should not be overlooked.

The above paucity of data and lack of consensus is in stark contrast with the adult oncology cohort where studies have quoted figures ranging from 27% to 75% mortality due to lower respiratory tract infection (LRTI) attributed to HPIV3 ^19^. This highlights the importance of further study and surveillance of the impact of HPIV3 in the pediatric cohort. This is particularly salient as currently, despite significant advances in the field, there is no vaccine or treatment for HPIV3, putting the onus on prevention and infection control. In this paper we present a highly robust system that allows both the incorporation of molecular and epidemiological data using Structured Query Language (SQL) ^20^, as well as a clear visualization tool based on Konstanz Information Miner (KNIME) version 3.3.2 ^21^. The latter is an open source tool that has been successfully used in the pharmaceutical and food industries and clinical epidemiology analysis ^22– 25^.

## Methods

### Ethical considerations

Ethical approval 12/EE/0069 including use of anonymised or link-anonymised patient data for outbreak investigation.

### Clinical details of the patients

The clinical details of the patients involved, including their underlying diagnosis, any other organisms isolated and potential impact of the infection are summarized in Table 1. It is important to note that although all patients involved were under some degree of immunosuppression due to their underlying condition or treatment, one was post transplant. The most common underlying diagnosis (6/15) was acute lymphocytic leukemia (ALL) and other blood cancers such as Non Hodgkin’s lymphoma, anaplastic large cell lymphoma (ALCL) and acute myeloid leukemia (AML) accounting for 3 more cases. There were four cases of CNS malignancy, one of a non-haematological malignancy and one post transplant. The most common cause of admission (8/14) was febrile neutropenia, which is defined as pyrexia with a low (<0.5 x 10 ^9^) neutrophil count ^26^, the rest were either routine admissions or admissions otherwise unrelated to infection: one relapse and one new diagnosis. Standard infection control measures include isolation of symptomatic patients, even before laboratory diagnosis is available, where possible or cohorting if single rooms are not available. Daily communication between the infection control team and senior unit nursing staff, informed by any virology results was used to determine the correct management for each potentially infected patient.

**Table 1.** Clinical details of patients involved in the outbreak. This table summarises the clinical details including the underlying diagnosis as well as other microorganisms isolated for patients 1-9,11,12,13,14 and 15. Patient 10 had a genetically different strain of human parainfluenza viruses type 3 (HPIV3) and was therefore not included in the final analysis. There was only one fatality (patient 6) that was unrelated to infection with HPIV3. Negative numbers of days indicate that the patient became symptomatic before the admission when they were diagnosed for HPIV3. This does not exclude a potential infection prior to this admission (see Figure 5).

Lab ID	Underlying diagnosis	Reason for admission	Interval between AD and SD (days)	Interval between AD and SOD (where available) (days)	Interval between SOD (where available) and SD (days)	Potential nosocomial infection?	Potential cause of admission/prolonged stay?	Other organisms isolated
1	CNS tumour	febrile neutropenia	1	-2	3	N	Y	rhinovirus
2	CNS tumour	febrile neutropenia	2	0	2	Y	Y	adenovirus
3	CNS tumour	febrile neutropenia	4	0	4	N	Y	rhinovirus
4	AML	new diagnosis	15	13	2	Y	N	
5	ALL	elective	0			N	Y	Gram +ve cocci *
6	ALL	febrile neutropenia	33			N	N	
7	ALCL	febrile neutropenia	1	0	1	Y	Y	rhinovirus
8	ALL	elective	1	0	1	N	N	
9	ALL	relapse	18	11	7	Y	N	
10	post- transplant	post- transplant	253			N	N	
11	ALL	febrile neutropenia	2			Y	Y	rhinovirus
12	non-malignant haematology disorder	elective	0			N	N	
13	Non Hodgkin's lymphoma	elective	0	-3	3	Y	Y	
14	CNS tumour	febrile neutropenia	4			Y	Y	rhinovirus
15	ALL	febrile neutropenia	0			N	Y	rhinovirus

* blood culture AD = admission date SD = sample date SOD=symptom onset date CNS=central nervous system AML=Acute myeloid leukemia ALL = Acute lymphoblastic leukemia ALCL = Anaplastic large cell lymphoma

### Respiratory virus diagnosis

All patients were diagnosed by routine respiratory diagnostic PCR on upper respiratory tract samples (either swabs or naso-pharyngeal aspirates). The validated multiplex PCR diagnostic panel used includes the following common respiratory viruses: influenza A and B, RSV, enterovirus, rhinovirus, human metapneumovirus (HMPV), adenovirus and human parainfluenza viruses. Turnaround time for results is generally 48 hours. The samples were identified as belonging to human parainfluenza virus 1 and 3 by the respiratory diagnostic panel and subsequently subtyped further by the HPIV subtyping PCR panel.

### Outbreak identification and sample collection

The number of samples tested positive for HPIV3 was extracted from the local databases from January 2011 until end of August 2017. Positive samples were deduplicated by each patient's unique hospital number, their date of birth, and if they had a positive sample within 72 days of a previous positive sample ^8^. The data was visualized in GraphPad Prism v 6.00 for Mac, GraphPad Software, La Jolla California USA. Epidemiological trends were compared between the number of positive samples for the six adjoining geographical areas covered by the laboratory and those for the hospital alone for years 2014–2017 and the pediatric oncology unit for the same dates up to and including August 2017. Once the outbreak was identified, samples from the 15 outbreak cases and 15 non-outbreak parainfluenza cases were collected for further analysis.

All samples were link-anonymised for laboratory work and patient demographics and immunosuppression status were retained where possible. Further patient information including patient movement, symptom onset, other organisms isolated and clinical outcome were extracted from the hospital electronic patient record.

### Primer design

**Figure 1.**
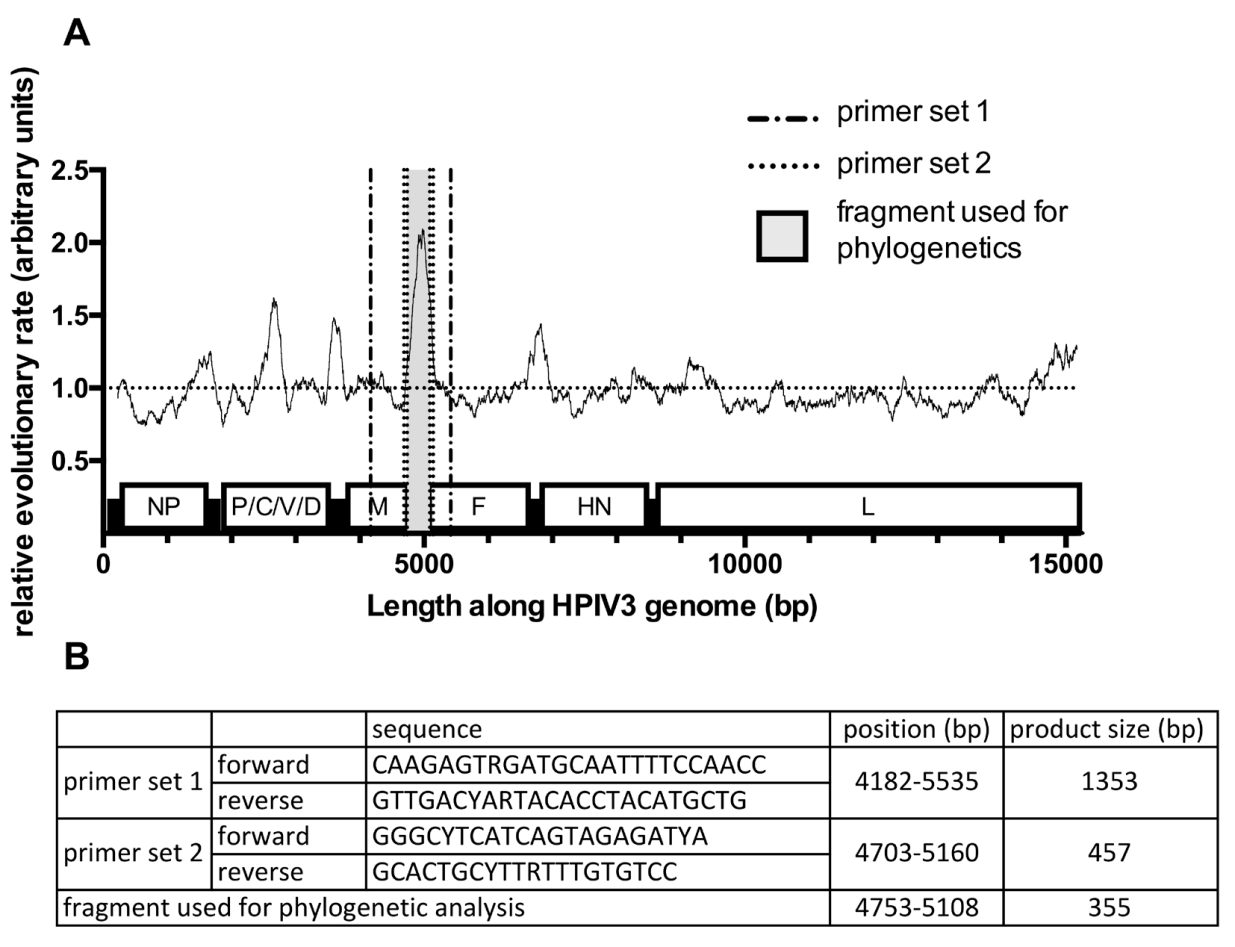
Hypervariable region of human parainfluenza viruses type 3 (HPIV3) and primer design. Figure **1A** shows the mean (relative) evolutionary rate per site with a window of 200. These rates are scaled such that the average evolutionary rate across all sites is 1. This means that sites showing a rate <1 are evolving slower than average and those with a rate >1 are evolving faster than average. These relative rates were estimated under the General Time Reversible model (+G+I) in MEGA7. The analysis involved 56 nucleotide sequences. The position along the HPIV3 genome is shown on the X axis. Figure **1B** summarises the sequences and positions of the two primer pairs for nested amplification of the hypervariable region as well as the segment used for subsequent phylogenetic analysis. The positions of these are shown diagrammatically in **1A**.

Thirty four HPIV3 whole genome sequences from diverse backgrounds and twenty UK based sequences from a companion paper were aligned in UGENE v 1.26.0 using the Muscle algorithm ^27^. A hypervariable region suitable for phylogenetic analysis (bp 4703-5160 KM190938.1) was identified in a companion paper ^28^. Two nested sets of primers flanking this region were designed ( Figure 1) and used for subsequent amplification.

### Extraction and amplification

Total RNA from samples was extracted using the GenElute Mammalian Total RNA Miniprep kit (Sigma, catalogue number RTN350) according to the manufacturer’s guidelines. Amplification was carried out using nested PCR with primers summarized in Figure 1. The first cycle was carried out with Superscript III One-step RT-PCR System with Platinum Taq High Fidelity from Invitrogen. The reverse transcription (RT) step was performed at 50°C for 30 min. This was followed by a 2min denaturation step at 94°C, and 35 cycles of denaturation (94°C for 15s), annealing (55°C for 30s) and extension (68°C 3min 30s). After the final extension step (68°C for 5min) the reaction was held at 4°C. The second cycle was performed with Taq DNA polymerase (Invitrogen) with the same cycling conditions without the RT step (5μl of product of the first cycle amplification was used as template).

Following two cycle amplification the products were ran on a 1% agarose gel for confirmation and cleaned following the Epoch Life Science Quick Protocol for EcoSpin All-in-one Mini Spin Columns. (catalogue number 1920-050/250) Rhinovirus genotyping was attempted for patients 1,3,7,11,14 and 15 and was conducted according to a previously published protocol ^29^. In brief, the protocol specifies two separate RT-PCR assays, targeted to the VP4/VP2 and 5′ UTR (untranslated region) regions of the rhinovirus genome. The VP4/VP2 assay constitutes a one-step RT-PCR reaction followed by a nested-PCR reaction. The 5′ UTR assay is a one-step RT-PCR reaction. The amplification products were then purified and sequenced by Sanger sequencing. For full details including primers used, please see 29.

### Sequencing and alignment

The amplicons were subsequently sequenced by Sanger sequencing and the contigs were assembled to a reference genome using Sequencher 5.4 from Genecodes. For HPIV3 a consensus sequence of the hypervariable region was extracted using UGENE v 1.26.0 ( Figure 1) as described in the companion paper ^28^. It was subsequently trimmed by 50 bp and 52 bp at the 3′ and 5′ prime ends respectively. The resulting fragment had good quality coverage in both directions and was therefore deemed to be suitable for further phylogenetic analysis. For rhinovirus, the alignments were done to the consensus sequence of published rhinovirus sequences KX610685; KY131965; KY645964; MF422576; MF422577; MF422578; MF422580; MF422581, as described previously ^28^. The consensus was extracted using alignments in UGENE v 1.26.0 using the Muscle algorithm ^29^.

### Phylogenetics

Phylogenetic analysis was performed as described in the companion paper ^28^. In brief the most suitable phylogenetic substitution model was selected using the JModelTest 2.0 Software ^30^ for Maximum likelihood analysis using Molecular Evolutions Genetics Analysis (MEGA) software MEGA v 7 ^31^ and marginal likelihood estimation using path sampling (PS) and stepping stone sampling (SS) was used for Bayesian Markov Chain Monte Carlo (MCMC) inference using BEAST v 1.8.4 ^32– 35^. Phylogenetic trees were visualized and edited using FigTree v 1.4.3.

### Epidemiology

15 patients involved in the outbreak were identified as described above. Data pertaining to patient admissions was collected between 08/05/2017 and 31/08/2017. In order to visualize the respective locations of patients, a timeline incorporating admission dates, symptom onset and first confirmed HPIV3 positive sample was constructed in GraphPad Prism version 6.00 for Mac, GraphPad Software, La Jolla California USA. Data was then organized in an SQL database. For the purposes of the model, the susceptibility period was defined as 1–7 days prior to symptom onset and the infectious period as 4 days prior to symptom onset. The patient was then considered infectious for the remaining duration of the outbreak due to prolonged asymptomatic shedding exhibited by this patient cohort. Symptom onset was defined as any viral respiratory symptom in either the upper or lower respiratory tract including exacerbations of previously known respiratory conditions. Where no symptom date was available the date of the first positive HPIV3 sample was used. The locations of patients included the pediatric oncology ward involved in the outbreak, the pediatric day unit (PDU) and the pediatric intensive care unit (PICU). Patients were in isolation at various times during their stay and freely ambulant on the wards during others. The data was subsequently visualized in KNIME 3.3.2 FoodChain-Lab ^24^.

## Results

### Outbreak identification and sample selection

**Figure 2.**
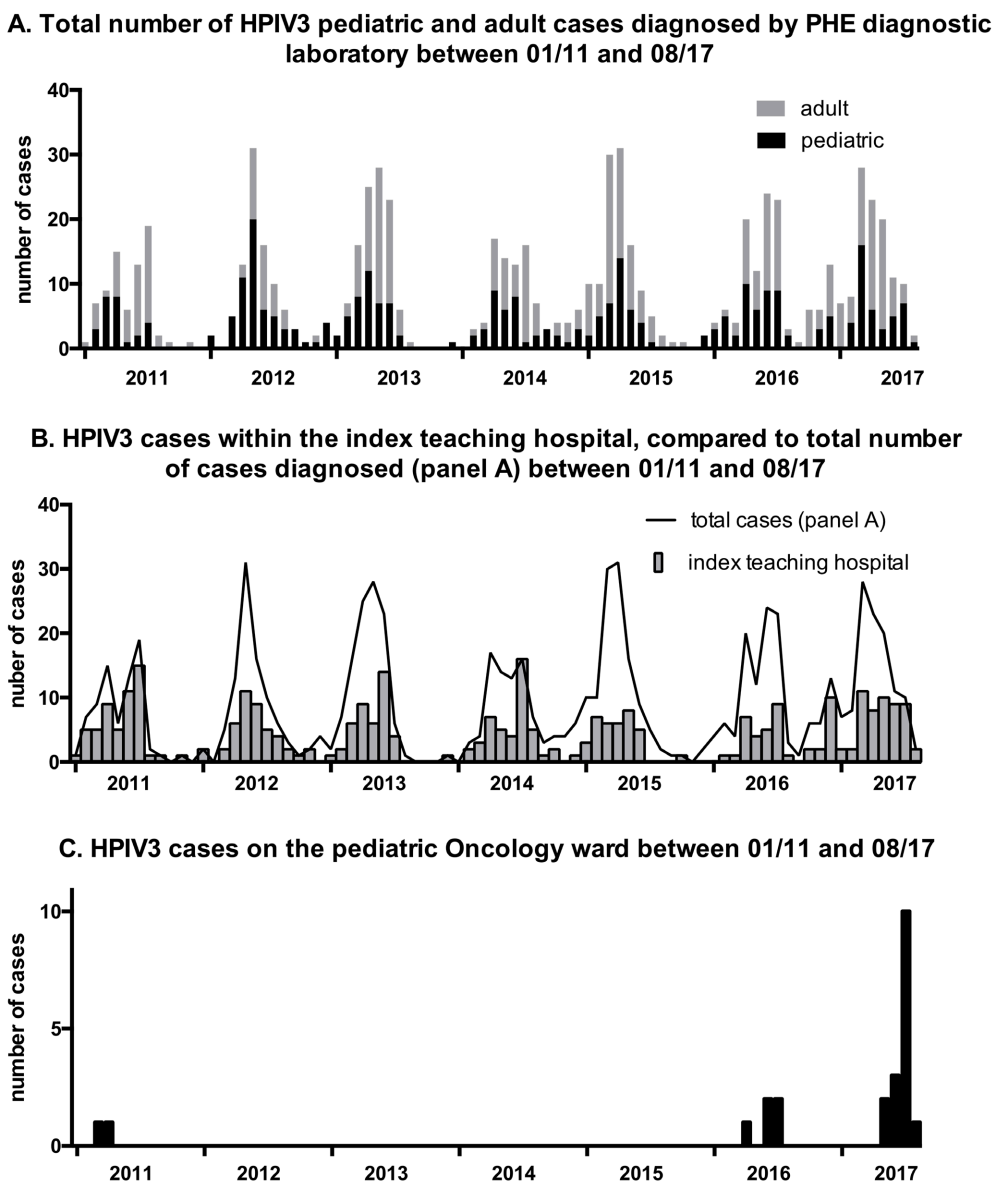
Epidemiological incidence of human parainfluenza viruses type 3 (HPIV3) and outbreak identification. The total number of HPIV3 cases diagnosed by the Public Health England (PHE) diagnositic laboratory, at the index teaching hospital, subdivided by adult and pediatric cases during the period Jan 2011 to August 2017 is shown in **A**. Number of cases for the same time period, geographically located within the index teaching hospital itself, compared to the total number of cases diagnosed are shown in **B**. Cases on the pediatric oncology ward within the same time period are shown in **C**.

The number of positive cases identified by the PHE laboratory at the index teaching hospital between the years 2014–2017 is summarized in Figure 2a. Expected seasonal fluctuations in HPIV3 prevalence, with peaks in late spring and summer were observed. No unusual activity pointing to a potential outbreak was identified. This was confirmed when the number of total HPIV3 cases in the index teaching hospital was compared to the number of cases within the full geographical area covered by the laboratory ( Figure 2b). However the number of cases observed in the pediatric oncology unit clearly reflected a peak centered in June–August 2017 ( Figure 2c). This was seen to exceed the usual seasonal fluctuations observed on this ward during previous seasons and an outbreak was suspected on the 14 ^th^ of July 2017. 

### Molecular analysis of the outbreak

**Figure 3.**
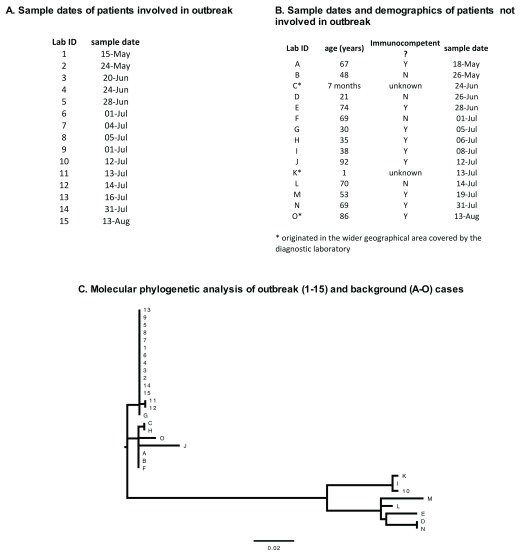
Outbreak (1-15) and non-outbreak (A-O) sample details and molecular phylogenetic analysis by Maximum Likelihood Method. Panel **A** summarises the dates of first human parainfluenza viruses type 3 (HPIV3) positive samples for all patients (1-15) involved in the outbreak. Demographics of patients not involved in the outbreak (A-O) are shown in panel **B**, those marked with an asterix originated in the wider geographical area rather than at the index teaching hospital. Panel **C** shows the molecular phylogenetic analysis of all the samples. The evolutionary history was inferred by using the Maximum Likelihood method based on the Tamura-Nei +I model and 1000 bootstrap repetitions. The tree is drawn to scale, with branch lengths measured in the number of substitutions per site with the legend shown. There were a total of 351 positions in the final dataset. Evolutionary analyses were conducted in MEGA7.

Consequently an attempt was made to confirm nosocomial transmission by genotyping the HPIV3 strains in this cluster. 30 cases were selected for molecular analysis, where 15 constituted the suspected outbreak and 15 were control cases unrelated to the outbreak. The pertinent clinical information for all cases is summarized in Figure 3a and b. All patients involved in the outbreak were coded 1-15. These were all pediatric patients in the oncology unit. Samples obtained for background phylogenetic information were coded A-O. These were drawn from a wider demographic in terms of age (range 7 months – 92 years), geographical location and immunosuppression status. It is of note that among the unrelated cases, the majority of patients (9/15) were not known to be either immunocompromised or immunosuppressed.

The maximum likelihood tree was constructed using the Tamura-Nei model with invariant sites ^36^ ( Figure 3c) using MEGA7, as detailed in the methods. Phylogenetic analysis indicated that 12/15 outbreak strains were genetically identical, with strains 11 and 12 differing from the rest of the cohort by one nucleotide, A182C. For confirmation, strains 11 and 12 were sequenced twice to exclude sequencing errors. Background strain G was shown to be identical to the main outbreak strain. This confirmed that this strain was circulating in the community rather than being unique to this outbreak. Outbreak sample 10 was seen to cluster separately from other cases involved in the outbreak. This was consistent with the clinical history of patient 10, who had been an inpatient for a number of months prior to this incident ( Table 1) and has had no points of contact with any of the other cases. Other non-related strains were shown to form two separate clusters, one being closely related to the outbreak strains (approximately 98% conformity with the outbreak strain) and the other more distinct (approximately 87% conformity with the outbreak strain). 

As rhinovirus was identified as a common secondary pathogen (6/15 cases), genotyping of rhinovirus was attempted using an established protocol ^26^. To this end the VP4/VP2 region of the rhinovirus genome sequence was amplified and aligned successfully for patients 7, 11 and 3. All strains of the virus were found to be different and therefore no evidence of dual infection transmission was found in these cases.

### Phylogenetic analysis in the context of other strains of HPIV3

**Figure 4.**
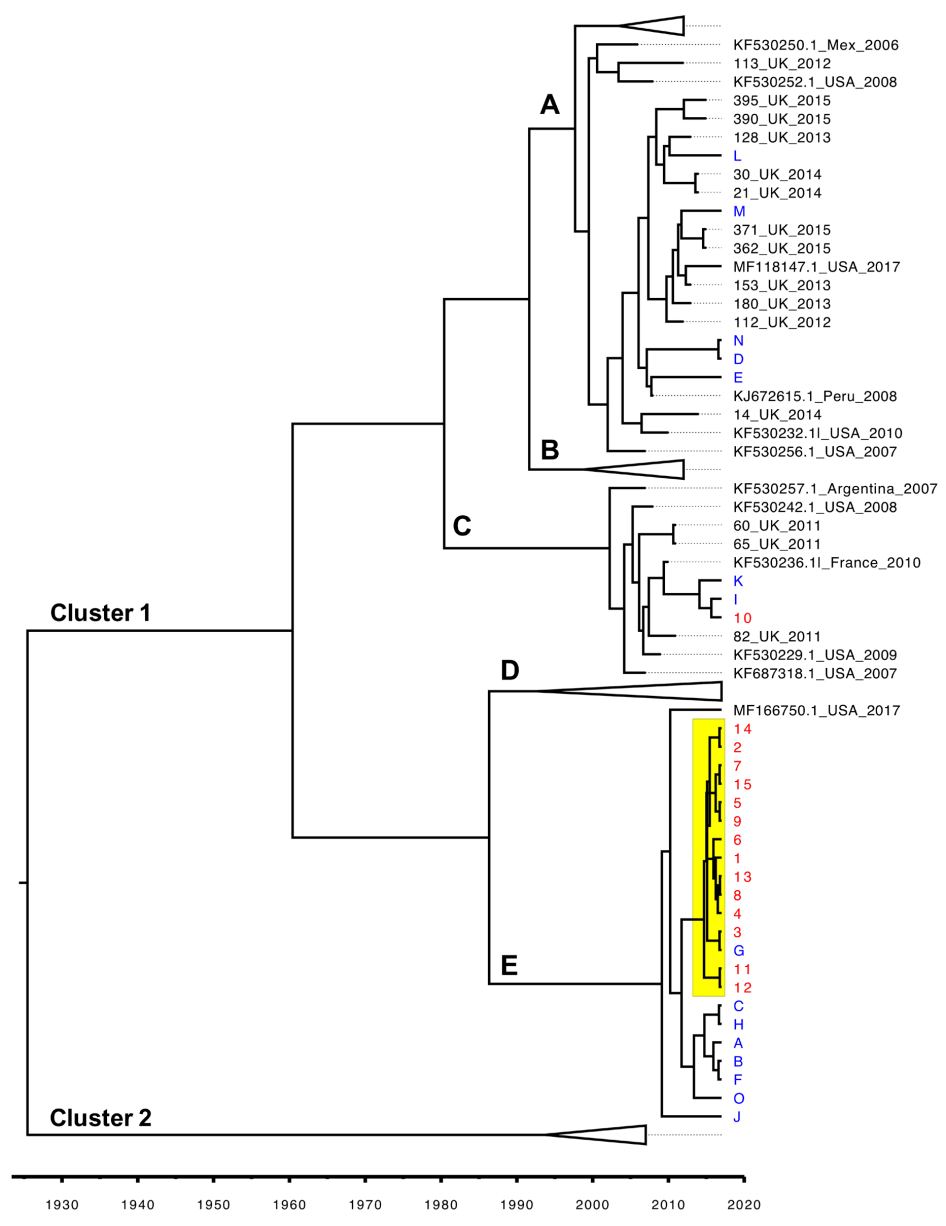
Molecular analysis of outbreak (1-15 red) and non-outbreak (A-O blue) strains in the context of historically circulating strains of human parainfluenza viruses type 3 (HPIV3). The evolutionary history was inferred using Bayesian Phylogenetics based on the Tamura-Nei +I model using BEAST v1.8.4 with a strict clock and constant coalecent prior. The MCMC length was 10,000,000. Convergence was assessed with Tracer (effective sample size >200). Inferred dates of strain emergence (in years) are shown in the figure legend. Clusters were defined with Automatic Barcode Gap Discovery. Clusters B and D have been collapsed for ease of visualization. All outbreak strains (1-15) are outlined in red and all background non-outbreak strains (A-O) are in green. The outbreak cluster is highlighted in yellow. The full details of UK circulating strains can be found in the companion paper ^28^.

Having confirmed a point-source outbreak, the sequences obtained were analyzed in the context of other historically circulating strains to establish whether the outbreak was caused by a newly emerging strain. To this end a Bayesian phylogenetic tree including the hypervariable region from the 30 strains identified above, as well as the 54 strains used for primer design, as detailed in the methods, was constructed using BEAST v1.8.4 ( Figure 4). This analysis confirmed a point source outbreak and served to illustrate that the outbreak strain was closely related to USA strain MF166750 2017, which was identified as a potential emerging strain in a the companion paper. 7/15 of strains (A, B, C, F, H, O, J) not involved in the outbreak were shown to be closely related to the outbreak strain. The remaining 7 background strains and outbreak strain 10 were found in subclusters 1A (L, M, N, D, and E) and 1C (K, I and 10) and demonstrate the diversity of HPIV3 strains circulating within a given time period.

**Figure 5.**
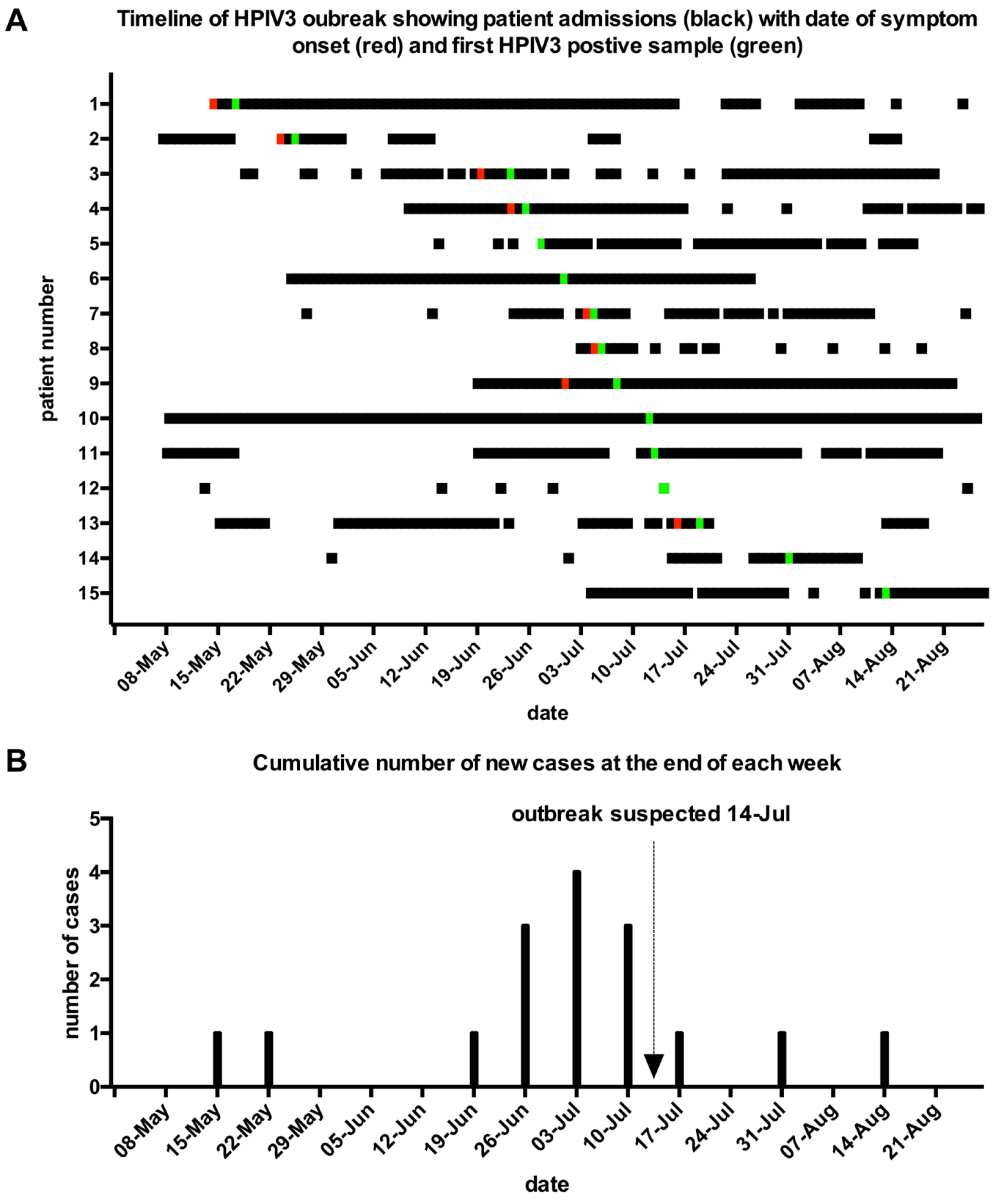
Timeline of patients’ admissions to the hospital including the date ( **A**) and cumulative number of new cases of human parainfluenza viruses type 3 (HPIV3) at the end of each week ( **B**) during May–August 2017. Panel **A** shows the movements in and out of hospital of 15 patients (coded 1-15 on the y axis) involved in the outbreak during the period encompassing a week before the first confirmed positive sample (15 May; patient 1) and until two weeks after the resolution of the outbreak in the end of August (last positive sample date 13 August; patient 15). Corresponding dates are shown on the x axis. Patient’s inpatient admissions are shown in black. Where a day visit was recorded in the notes, it is reflected as an admission of 1 day on the timeline. Symptom onset dates, if available, are shown in red. Dates of confirmed positive samples are shown in green. Panel **B** shows the cumulative number of new cases of HPIV3 at the end of each week for the same period. The date that the outbreak was recognized (14 July) is marked with an arrow.

### Epidemiological analysis – timeline

After identifying the cluster as a point source outbreak the potential transmission routes between patients were investigated. First an attempt was made to identify the overlap of patients in space and time, to this end a timeline was constructed based on admission dates, symptom onset and first positive sample date for the patients involved ( Figure 5A). This clearly demonstrated a significant temporal overlap between patients involved in the outbreak as well as potential delays between symptom onset and a sample being taken where available (8/15 cases). The number of cumulative new cases at the end of each week is shown in Figure 5B, together with the date when the outbreak was suspected. It is clear that the outbreak was identified as the number of cases was already on the decline and therefore the window for meaningful intervention had passed.

### Epidemiological analysis – transmission

In order to map the potential transmission of infection, further data including the exact patient location, was then extracted and summarized in SQL together with the defined periods of potential susceptibility, infectivity and phylogenetic plausibility. Based on molecular data, Patient 10 was identified as not part of the outbreak. Patients 11 and 12, that had a strain that differed by one identical nucleotide from the main outbreak strain, remained as part of the outbreak. This was because the possibility of an original infection by the main outbreak strain or the acquisition of their unique strain from another patient could not be excluded. It was however considered impossible for them to infect another patient with a strain that lacked this unique nucleotide difference.

**Figure 6.**
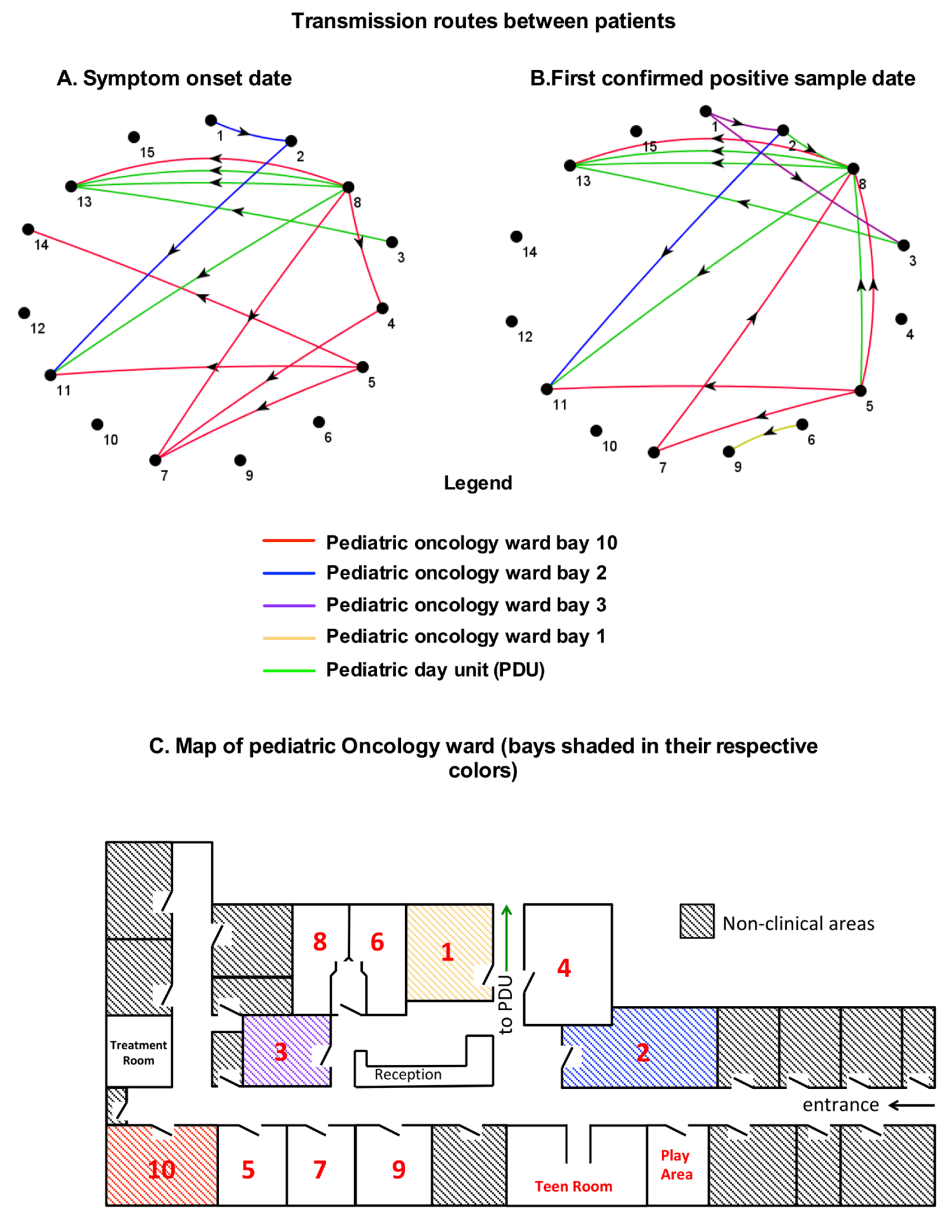
Inferred infection transmission routes between patients. Transmission routes between patients have been analysed with SQL and visualized using KNIME 3.3.2. Patient location, as well as symptom onset date, where available ( **A**) and first positive human parainfluenza viruses type 3 (HPIV3) sample date ( **B**) were used. All phylogenetically implausible connections have been removed. The connectors between patients are color coded according to patient location as shown in the legend and the direction of the arrow conforms to the direction of infection spread. The map of the unit with locations shaded in corresponding colors is shown in panel **C**.

KNIME visualization for potential transmissions using the date of symptom onset and confirmed HPIV3 positive sample is summarized in Figure 6. KNIME analysis by symptom onset date ( Figure 6a) has identified 12 phylogenetically plausible transmission events, linking 10/14 patients involved in the outbreak, and identifying a potential source of infection in 6/14 cases. Analysis by first positive sample date identified 15 phylogenetically plausible transmission events, linking 10/14 patients and identifying a potential source of infection in 7/14 patients. It is interesting to note that although these highly complex patients were periodically admitted onto a number of different wards, the pediatric oncology unit (ward and day unit) were the main hotspots for transmission. It is also of interest that despite extensive data mining and the flexibility of the model, it was possible neither to connect all the patients involved, nor identify a chain of infection from an index case. This indicates that an external source of infection, that has not been accounted for within the model, was involved.

### Clinical background and impact

Having established the molecular and epidemiological nature of the outbreak, an attempt was made to assess the potential clinical impact on the patient cohort in question. Of the 14 cases, identified as part of the outbreak, there was one fatality, which was not linked to the HPIV3 infection. It is however important to note that in 9/14 cases the infection with HPIV3 was identified as a potential cause for either an admission or extended stay. The most common additional organism isolated was rhinovirus and this reflects the common occurrence of this virus within the pediatric patient cohort.

## Discussion

In this study we have presented an outbreak involving 14 cases of HPIV3 on a pediatric oncology ward in a major teaching hospital. As reported in the literature by others, this nosocomial outbreak was not identified contemporaneously ^8, 9^. The nature of the outbreak was, to an extent, masked by the normal epidemiological pattern of HPIV3 involving late spring and summer time peaks ^3^ ( Figure 2). Seasonal bias is a well-recognized confounder in public health reporting. Outbreak detection algorithms usually include trend adjustments for seasonality ^37– 39^. Moreover, failures to identify outbreaks have previously been described in viral diseases with a strong seasonal bias ^40, 41^. In this case, root cause analysis identified failure of data integration systems and processes to facilitate rapid communication to allow the possibility of outbreak detection. To this end a respiratory virus tracking system using CHEQS software has since been incorporated into normal clinical practice to allow cumulative data collection and visualization of trends by time and geographical location.

Previous HPIV3 genomic analysis in the context of outbreaks has been conducted by amplifying the HN part of the genome ^8^ as well as a fragment of the hypervariable region as amplified in this study ^42, 43^. The protocol was originally attempted side by side with the protocol described in this study with the latter yielding much higher success rates. The temporal component was analyzed using Bayesian phylogenetics in the context of other currently and previously circulating strains of HPIV3 ( Figure 4). The phylogenetic analysis served to establish this as a point-source outbreak ( Figure 3) and the strain involved as newly emerging with some of the community strains closely related to it ( Figure 4). As this cluster of strains has only been identified in 2017, it could be argued that it could cause re-infections in otherwise healthy individuals. The above was supported by phylogenetic data: a case of the outbreak strain (case G, Figure 4) was identified in the wider community.

Other community strains (K, I, L, M, N, D and E, Figure 4) as well as the patient 10 strain have been classified as belonging to two different subclusters ( Figure 4), one of which, subcluster 1C, had been identified as early as 2011. The latter demonstrates the variety of circulating HPIV3 strains within any given time period with no clear geographical correlation. Although the newly emerging outbreak strain is clearly related to a strain originating in the USA in 2017, one cannot infer that these two strains are confined to UK and USA. Furthermore it cannot be discounted that strains 11 and 12 that differ by a single mutation A182C from the rest of the outbreak strains, have evolved separately from the outbreak strain. For the purposes of epidemiological analysis it was therefore only inferred that both of these patients have either been infected by a single source or that one had infected the other.

In the context of nosocomial outbreaks, symptom onset data is frequently not available and the date of first positive sample is used for all epidemiological modeling. Although sufficient for outbreak tracing, this can potentially underestimate the impact of early symptom identification and prompt screening on outbreak prevention. To this end, this data, together with data on patient admission was collected where possible ( Figure 5). Subsequently, transmission models involving both dates, if available, were included in this study ( Figure 6). Twelve transmission events, linking 10 patients were identified using the symptom onset date, whereas 15 potential transmission events were identified by the model using the positive sample date. Neither model could be used to pinpoint a clear infection chain from the index case, and an infection source was only identified in 5 cases for symptom onset date analysis and 6 cases for confirmed positive case date analysis ( Figure 6). It is important to note that data on symptom onset was only available in 8 out of 14 cases involved in the outbreak and this was further confounded by secondary infections ( Table 1). Overall within the limitations of the data available, the current analysis identified 3 potential transmission events and one potential infection that could have been prevented if screening were carried when symptoms were first identified.

It is also important to note that neither model could successfully identify transmission between all the patients involved in the outbreak. We could therefore surmise that either another source of infection or other points of contact between patients were present. Cases of health care workers contributing to nosocomial outbreaks are well documented ^44– 47^. Although stringent infection control policies are usually in place, transmission from a member of staff cannot be excluded.

The clinical impact of this outbreak is difficult to assess. No fatalities that could be related to HPIV3 infection were identified. A recent investigation of an HPIV3 outbreak in a similar setting has also failed to identify any fatalities associated with this virus ^8^. However, HPIV3 was recognized as a potential cause of an admission or prolonged stay in 9 out of 14 cases, although in 8 out of 9 cases a potential secondary pathogen was also identified, emphasizing the potential importance of dual infections ( Table 1). Rhinovirus was a notable secondary viral respiratory pathogen, isolated in six of the cases. It is a highly prevalent virus in the pediatric cohort with a non-seasonal occurrence and especially common in dual infections ^11^. Rhinovirus genotyping was successful in 3 of the cases but did not identify any instances of dual infection transmission in this study, although the incidence was broadly in keeping with some previous reports ^16^.

In conclusion in this study we have presented a cluster of HPIV3 cases in a pediatric oncology unit in a major teaching hospital. This cluster was subsequently identified as a point-source outbreak involving 14 out of 15 cases and as in other reported outbreaks in pediatric cancer units, was only identified retrospectively ^8, 9^. Root cause analysis has identified a number of factors that contributed to inability to identify the outbreak. This was addressed by introducing a new respiratory virus tracking software.

The outbreak strain was recognized as a new emerging strain. The epidemiological transmission analysis has highlighted the importance of early identification and screening of both patients and staff. Potential spread by staff was inferred from transmission hotspots identified by the epidemiology transmission model. The challenge of managing staff with minimal respiratory symptoms in a pediatric oncology unit has not been studied sufficiently and the literature in this area is sparse. Until this outbreak, it was not policy to screen staff – this is now under review. The main clinical impact of the outbreak in this study was in the number of increased admissions and hospital stay. Further analysis of the importance of HPIV3 within the pediatric oncology cohort, is required to evaluate the pertinence of these investigations in this context. 

## Data availability

All HPIV3 sequences have been uploaded to NCBI with the following references and accession numbers:

HPIV3/UK/ **1**/15/05/2017      MH699933

HPIV3/UK/ **2**/24/05/2017      MH699934

HPIV3/UK/ **3**/20/06/2017      MH699935

HPIV3/UK/ **4**/24/06/2017      MH699936

HPIV3/UK/ **5**/15/05/2017      MH699937

HPIV3/UK/ **6**/01/07/2017      MH699938

HPIV3/UK/ **7**/04/07/2017      MH699939

HPIV3/UK/ **9**/01/07/2017      MH699941

HPIV3/UK/ **13**/16/07/2017    MH699942

HPIV3/UK/ **14**/31/07/2017    MH699943

HPIV3/UK/ **11**/13/07/2017    MH699944

HPIV3/UK/ **12**/14/07/2017    MH699945

HPIV3/UK/ **15**/13/08/2017    MH699946

HPIV3/UK/ **10**/12/07/2017    MH699947

HPIV3/UK/ **A**/18/05/2017      MH699948

HPIV3/UK/ **B**/26/05/2017      MH699949

HPIV3/UK/ **C**/24/06/2017      MH699950

HPIV3/UK/ **D**/26/06/2017      MH699951

HPIV3/UK/ **E**/28/06/2017      MH699952

HPIV3/UK/ **F**/01/07/2017      MH699953

HPIV3/UK/ **G**/05/07/2017      MH699954

HPIV3/UK/ **H**/06/07/2017      MH699955

HPIV3/UK/ **I**/08/07/2017       MH699956

HPIV3/UK/ **J**/12/07/2017       MH699957

HPIV3/UK/ **K**/13/07/2017      MH699958

HPIV3/UK/ **L**/14/07/2017      MH699959

HPIV3/UK/ **M**/19/07/2017     MH699960

HPIV3/UK/ **N**/31/07/2017      MH699961

HPIV3/UK/ **O**/13/08/2017     MH699962

All uploaded sequences can be accessed together via PopSet: 1470015972

Raw sequences and annonymised epidemiological data has been deposited on Open Science Framework. Dataset 1: Unrecognised Outbreak: Human Parainfluenza Virus Infections in a pediatric oncology unit. A new diagnostic PCR and virus monitoring system may allow early detection of future outbreaks https://doi.org/10.17605/OSF.IO/X4ZYC ^48^

License: CC0 1.0 Universal
