# Supplementary material for: Unrecognised Outbreak: Human parainfluenza virus infections in a pediatric oncology unit.  A new diagnostic PCR and virus monitoring system may allow early detection of future outbreaks
Source: Wellcome Open Res. 2018 Sep 19;3:119. [Version 1] doi: 10.12688/wellcomeopenres.14732.1 (PMC6338131; doi:10.12688/wellcomeopenres.14732.1)
Supplement: Peer review history

# Peer review: Referee response for version 1

**DOI:** 10.21956/wellcomeopenres.16050.r34450

**Published:** 2019-01-15

**Authors:** Eric J. Chow

**Affiliations:** 1 Epidemic Intelligence Service, Centers for Disease Control and Prevention (CDC), Atlanta, GA, USA

Thank you for the opportunity to review this manuscript. In their research article entitled “Unrecognised Outbreak: Human parainfluenza virus infections in a pediatric oncology unit. A new diagnostic PCR and virus monitoring system may allow early detection of future outbreaks” the authors describe a cluster of human parainfluenza virus infection cases at their hospital. In writing up this case, the author highlight the potential life-threatening aspects of this type of outbreak especially among immunocompromised pediatric oncology patients. Nosocomial viral respiratory infections continue to be an underappreciated source of morbidity and mortality in hospitalized patients and the authors' paper adds to the literature highlighting its importance.

 The authors do an excellent job detailing out their discovery of the outbreak. I would like to know a little more information about their pediatric hospital (size, number of patients, tertiary care center?) which will help the generalizability of their findings. I personally would find some demographic information about the patients involved in the outbreak including age/gender which would help visualize those who were affected. Lastly, what is the typical incubation period for parainfluenza virus? 

I have read this submission. I believe that I have an appropriate level of expertise to confirm that it is of an acceptable scientific standard.

# Peer review: Referee response for version 1

**DOI:** 10.21956/wellcomeopenres.16050.r34422

**Published:** 2019-01-14

**Authors:** Darrell L Dinwiddie

**Affiliations:** 1 Department of Pediatrics, Clinical and Translational Science Center (CTSC), University of New Mexico Health Sciences Center, Albuquerque, NM, USA

The authors provide a high-quality retrospective investigation into a potential human parainfluenza virus 3 (HPIV3) outbreak in a pediatric oncology unit.  Through PCR and Sanger sequencing of a hypervariable region of the HPIV3 genome and phylogenetic analysis, the authors investigated 15 samples from the potential outbreak.

 Sequencing and phylogenetic analysis revealed that 12 HPIV3 outbreak samples were identical, 2 samples varied by only a single nucleotide from the cluster of 12, but were identical to each other, and 1 outbreak sample that varied significantly from the other 14 samples, but was phylogenetically grouped with non-outbreak samples. Sequencing of non-outbreak samples showed distinct phylogenetic clustering and higher genetic variability as compared to 14 of the 15 potential outbreak samples. Taken together, these results suggested a nosocominal outbreak of HPIV3 that consisted of at least 14 patients.

 It is notable that the majority of infections and transmission events (78%) occurred prior to the outbreak being suspected and staff were suspected to account for some of the transmission source of contact.  Furthermore, it is likely that the 14 cases is an under-representation of the outbreak since additional patients may have been missed. Thus, the authors suggest that this study highlights the importance of early identification and screening/surveillance of patients and staff.

 The authors attempted to assess the clinical impact of the outbreak, but the analysis was complicated by a high number of potential co-infections. Nonetheless, an infection or co-infection was recognized as a contributing factor in hospital admission or prolonged stay in the majority of patients from the outbreak.  A nice followup study might assess the clinical impact and costs of respiratory infections from HPIV3 and other pathogens as compared to infection control and surveillance/screening efforts.

I have read this submission. I believe that I have an appropriate level of expertise to confirm that it is of an acceptable scientific standard.

# Peer review: Referee response for version 1

**DOI:** 10.21956/wellcomeopenres.16050.r34290

**Published:** 2018-11-28

**Authors:** Richard Soutar

**Affiliations:** 1 Greater Glasgow and Clyde Health Board, Glasgow, UK; 2 Scottish National Blood Transfusion Service, Edinburgh, UK

This is a good paper with impressive molecular virology and epidemiology identifying the development of a Paraflu 3 outbreak in a paediatric haematology unit.

 The authors are to be congratulated in their retrospective analysis of the outbreak which gives significant insight into the event.

 I am sure this paper will be of interest to virologists, epidemiologists, public health, infection control and clinicians.

 However, from the clinical point of view many of the challenges remain, which the authors readily state: identification of patient symptoms, complexities of screening asymptomatic patients, implications of staff screening, whether IT systems can help identify out breaks early. Perhaps it was out with the scope of the paper but having mentioned ‘root cause analysis’ and ‘introduction of new respiratory virus tracking software’ it would have been good to have had these discussed in further detail.

 Otherwise the authors are to be commended on this detailed scientific examination of an important clinical problem.

I have read this submission. I believe that I have an appropriate level of expertise to confirm that it is of an acceptable scientific standard.
